# Target of rapamycin complex 2–dependent phosphorylation of the coat protein Pan1 by Akl1 controls endocytosis dynamics in *Saccharomyces cerevisiae*

**DOI:** 10.1074/jbc.RA117.001615

**Published:** 2018-06-12

**Authors:** Clélia Bourgoint, Delphine Rispal, Marina Berti, Ireos Filipuzzi, Stephen B. Helliwell, Manoël Prouteau, Robbie Loewith

**Affiliations:** From the ‡ Department of Molecular Biology and Institute of Genetics and Genomics of Geneva (iGE3), National Center for Competence in Research in Chemical Biology, University of Geneva, 1211 Geneva, Switzerland and; § Novartis Institutes for Biomedical Research, Novartis Campus, 4056 Basel, Switzerland

**Keywords:** target of rapamycin (TOR), signal transduction, endocytosis, actin, membrane function, aminophospholipid flippase, Ark1/Prk1 family, Fpk1, in vitro kinase assay, Pan1

## Abstract

Target of rapamycin complex 2 (TORC2) is a widely conserved serine/threonine protein kinase. In the yeast *Saccharomyces cerevisiae*, TORC2 is essential, playing a key role in plasma membrane homeostasis. In this role, TORC2 regulates diverse processes, including sphingolipid synthesis, glycerol production and efflux, polarization of the actin cytoskeleton, and endocytosis. The major direct substrate of TORC2 is the AGC-family kinase Ypk1. Ypk1 connects TORC2 signaling to actin polarization and to endocytosis via the flippase kinases Fpk1 and Fpk2. Here, we report that Fpk1 mediates TORC2 signaling to control actin polarization, but not endocytosis, via aminophospholipid flippases. To search for specific targets of these flippase kinases, we exploited the fact that Fpk1 prefers to phosphorylate Ser residues within the sequence R*X*S(L/Y)(D/E), which is present ∼90 times in the yeast proteome. We observed that 25 of these sequences are phosphorylated by Fpk1 *in vitro*. We focused on one sequence hit, the Ark/Prk-family kinase Akl1, as this kinase previously has been implicated in endocytosis. Using a potent ATP-competitive small molecule, CMB4563, to preferentially inhibit TORC2, we found that Fpk1-mediated Akl1 phosphorylation inhibits Akl1 activity, which, in turn, reduces phosphorylation of Pan1 and of other endocytic coat proteins and ultimately contributes to a slowing of endocytosis kinetics. These results indicate that the regulation of actin polarization and endocytosis downstream of TORC2 is signaled through separate pathways that bifurcate at the level of the flippase kinases.

## Introduction

In *Saccharomyces cerevisiae*, two genes encode the target of rapamycin (TOR)^3^ kinase, *TOR1* and *TOR2* (1, 2), and the encoded proteins assemble into two highly conserved complexes named TORC1 and TORC2 (3, 4). TORC1 is formed by either Tor1 or Tor2, whereas only Tor2 can assemble into TORC2. The antifungal and immunosuppressant drug rapamycin specifically inhibits TORC1, and this macrolide has been used extensively to dissect how TORC1 signals control cell growth via its regulation of various anabolic and catabolic processes.

The insensitivity of TORC2 to rapamycin is due to the Avo3 subunit that sterically occludes the rapamycin-binding domain of Tor2 (5). The lack of a chemical probe to acutely inhibit TORC2 has hampered the dissection of the pathways downstream of this essential kinase. Initial studies to interrogate TORC2 signaling relied on the use of nutrient-regulated promoters and temperature-sensitive alleles of *tor2* (6–12). These studies revealed that the major effector of TORC2 is the AGC-family kinase Ypk1, through which TORC2 regulates polarization of the actin cytoskeleton and clathrin-mediated endocytosis (2, 13). However, nutrient and temperature shifts are far from ideal experimental setups to study TORC2 as both shifts *per se* inherently affect TORC2 kinase activity. Furthermore, with these protocols, phenotypes associated with the loss of TORC2 activity take hours to appear, making it difficult to differentiate between direct signaling events and indirect affects due to chronic inactivation of TORC2.

To address these shortcomings, we previously identified NVP-BHS345 as an ATP-competitive inhibitor of both Tor1 and Tor2 and demonstrated that this compound specifically and rapidly inhibits the kinase activities of both TORC1 and TORC2 (14). Subsequently, we found that a point mutation in *TOR1* and in *TOR2* that changes methionines 2282 and 2286 to a threonine, respectively, renders TORC1 and/or TORC2 largely insensitive to NVP-BHS345 (15). Thus, in a *TOR1^M2282T^* (*TOR1^MT^*) background, NVP-BHS345 treatment leads to an acute loss of TORC2 activity only. Using this experimental setup, we found that TORC2-Ypk1 regulates actin organization and endocytosis via the flippase kinases Fpk1 and Fpk2 (13, 15).

Fpk1 phosphorylates Dnf1 and its paralog Dnf2 (16, 17), 4P-type ATPases that regulate bilayer asymmetry by translocating specific phospholipids from the exocytic to the cytosolic leaflet of the plasma membrane (18–20). Plasma membrane localization of Dnf1/2 requires interaction with Lem3 in the endoplasmic reticulum (21), and *lem3* mutants, like *dnf1 dnf2* mutants, present defective flipping of phosphatidylethanolamine as evidenced by hypersensitivity to Ro09-0198 (22) and its analog duramycin (23).

In the present study, we made use of a similar chemical genetic approach enabling the specific inhibition of TORC2 with a novel, significantly more potent ATP-competitive inhibitor called CMB4563. We found that deletion of *LEM3* partially suppresses the actin depolarization, but not the endocytosis arrest, normally observed upon TORC2 inhibition. This observation suggested that the Fpks have additional targets beyond the flippases, some of which we identified in an *in vitro* screen querying the ability of purified Fpk1 to phosphorylate recombinant peptides derived from yeast proteins containing the Fpk1 consensus motif. One hit from this screen, Akl1, was chosen for further characterization. Alk1 shares sequence homology to two other kinases, Ark1 and Prk1, known to regulate endocytic vesicle uncoating via phosphorylation of multiple coat proteins, including Pan1, Sla1, and Ent1 (24–26). We found that, upon TORC2 inhibition, Fpk1-mediated phosphorylation inhibits Akl1 kinase activity, resulting in a loss of coat protein phosphorylation and ultimately a delay in endocytosis kinetics.

## Results

### CMB4563, a potent inhibitor of the TOR complexes in budding yeast

**Figure 1.**
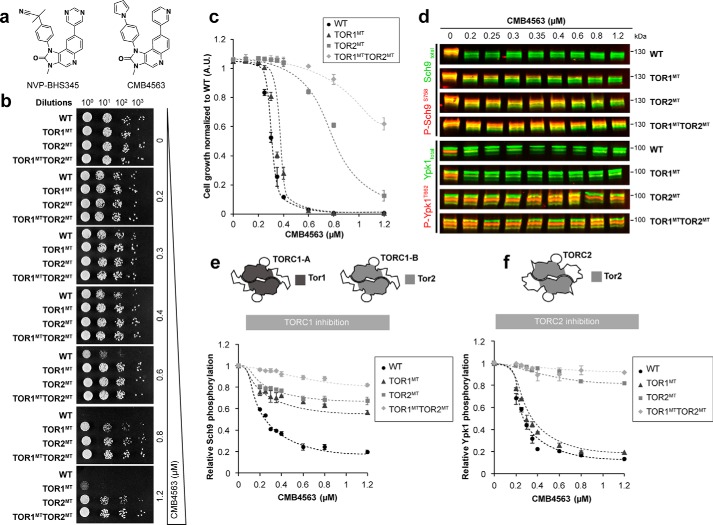
**Acute, specific chemical-genetic inhibition of TORC2.** *a*, structures of NVP-BHS345 (*left*) and CMB4563 (*right*). *b*, serial 10-fold dilutions of cells of the indicated genotype were spotted onto plates containing the indicated concentrations of CMB4563. *c*, growth inhibition of the indicated strains in liquid synthetic complete medium by CMB4563 assessed by high-resolution growth curves performed with biological triplicates. *Error bars* represent S.D. *d*, representative Western blot assessing Sch9^pSer-758^ and Ypk1^pThr-662^ phosphorylation as a respective measure of TORC1 and TORC2 activity following 12-min CMB4563 treatments of cells of the indicated genotypes. The corresponding split-channel version is in Fig. S1b. *e*, quantification (biological triplicates) of TORC1 inhibition as assessed in *d. Error bars* represent S.D. *f*, quantification (biological triplicates) of TORC2 inhibition as assessed in *d. Error bars* represent S.D. *A.U.*, absorbance units.

Previously, we characterized NVP-BHS345 as an inhibitor of TORC1 and TORC2 in yeast (15). Initial characterization of related molecules suggested that CMB4563 (Fig. 1*a*) also inhibits the two TOR complexes (Fig. S1a), prompting us to characterize this compound further. CMB4563 is toxic to WT cells grown either on solid medium, preventing colony formation at concentrations of 0.8 μm, or in liquid medium with an IC_50_ of 0.3 μm (Fig. 1, *b* and *c*). As observed previously with NVP-BHS345, expression of *TOR1^MT^* partially reduced CMB4563 toxicity, whereas expression of *TOR2^M2286T^* (*TOR2^MT^*) strongly reduced CMB4563 toxicity, consistent with Tor2 residing in both TORC1 and TORC2 (Fig. 1*c*). Coexpression of both *TOR1^MT^* and *TOR2^MT^* reduced toxicity even further. As expected, addition of CMB4563 to WT cells triggered dephosphorylation of both the TORC1 substrate Sch9 (Fig. 1, *d* and *e*, and Fig. S1b) and the TORC2 substrate Ypk1 (Fig. 1, *d* and *f*, and Fig. S1b). Expression of either *TOR1^MT^* or *TOR2^MT^* suppressed Sch9 dephosphorylation, whereas only *TOR2^MT^* suppressed Ypk1 dephosphorylation. TORC2 inhibition was rapid, occurring within a minute (Fig. S1c). Comparison with our previous study (15) shows that CMB4563 is ∼15 times more potent than NVP-BHS345 (IC_50_ values of 0.3 and 5 μm, respectively). We conclude that both TORC1 and TORC2 are the growth-limiting targets of CMB4563 in WT cells and that TORC2 is the growth-limiting target in *TOR1^MT^* cells.

### Fpk1 controls TORC2-dependent actin polarization and endocytosis via distinct effectors

**Figure 2.**
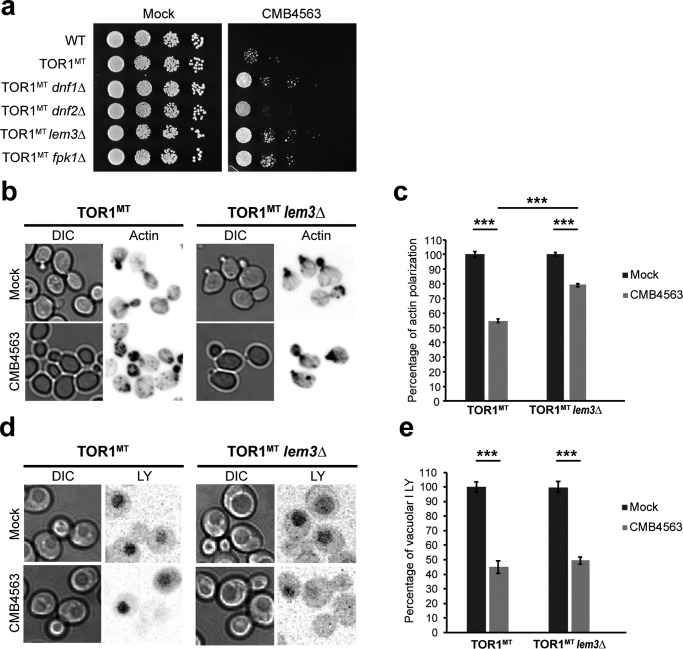
**TORC2 regulates actin polarization largely through the aminophospholipid flippases.** *a*, serial 10-fold dilutions of cells of the indicated genotype were spotted onto SCM plates containing or lacking 1.2 μm CMB4563. *b*, rhodamine phalloidin (actin) was used to stain the actin cytoskeleton in *TOR1^MT^* and *TOR1^MT^ lem3*Δ cells following no treatment or a 30-min treatment with 0.8 μm CMB4563. *c*, quantification of the extent of actin polarization as assessed in *b*. Plotted are the means normalized to the respective mock ±S.D. *Error bars* represent S.D. Statistical significance was determined using Student's *t* test; *p* < 0.0005 (***) based on biological triplicates with *n* > 100 cells/replicate. *d*, LY uptake was used to monitor fluid-phase endocytosis in *TOR1^MT^* and *TOR1^MT^ lem3*Δ cells following no treatment or a 30-min treatment with 0.8 μm CMB4563. *e*, quantification of the extent of Lucifer yellow accumulation in the vacuole as assessed in *d*. Plotted are the means normalized to the respective mock ±confidence interval (*p* value = 5%). *Error bars* represent confidence interval. Statistical significance was determined using Student's *t* test; *p* < 0.0005 (***) based on biological duplicates with *n* = 30 cells/replicate. *DIC*, differential interference contrast.

Previously, we had observed that codeletion of *FPK1* and *FPK2* modestly suppresses the toxicity and largely prevents the actin depolarization and endocytosis arrest otherwise observed upon TORC2 inhibition with NVP-BHS345 in *TOR1^MT^* cells (15). We repeated this experiment and found that deletion of *FPK1* alone was sufficient to modestly improve growth in the presence of CMB4563 (Fig. 2*a*). As Fpk1 phosphorylates and activates Dnf1 and Dnf2, we assessed whether deletion of *DNF1*, *DNF2*, or *LEM3* also conferred resistance to CMB4563. Deletion of any one of these three genes conferred partial resistance to CMB4563 (Fig. 2*a*). Then we assessed whether TORC2 regulates actin polarization and/or endocytosis via Lem3. Interestingly, we found that although *LEM3* deletion partially suppressed actin depolarization (∼80% of *lem3* cells possessed a polarized actin cytoskeleton after TORC2 inhibition compared with ∼50% of *LEM3* cells; Fig. 2, *b* and *c*) it failed to suppress the endocytosis defect as measured by the inability to accumulate Lucifer yellow (LY) in the vacuole (Fig. 2, *d* and *e*). These results confirm that the phospholipid flippases mediate TORC2-dependent signals to polarize the actin cytoskeleton (27) but show that other substrates mediate signaling to the endocytosis machinery.

### In vitro screen for new targets of Fpk1

**Figure 3.**
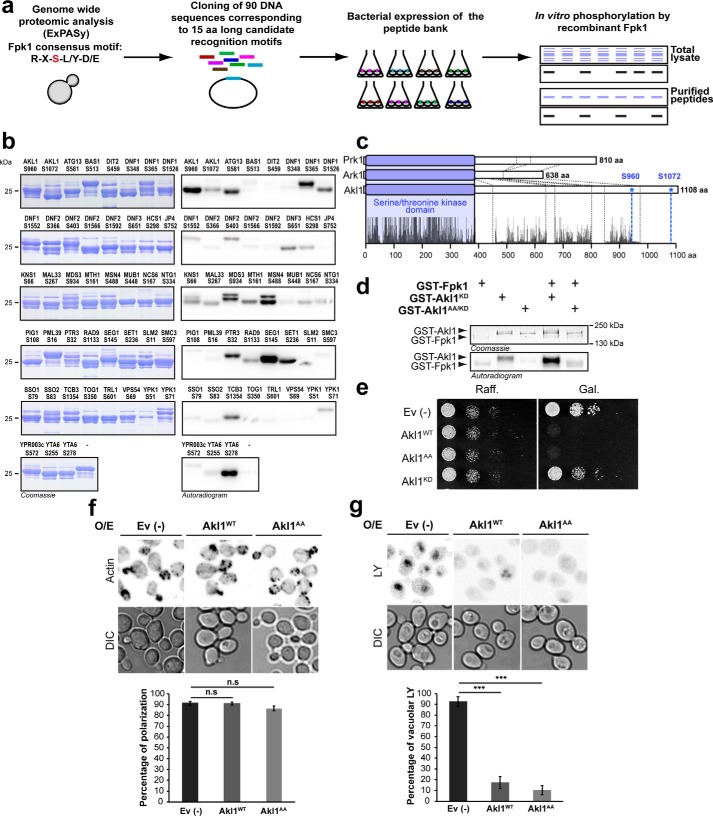
***In vitro* kinase assay screen for Fpk1 substrates.** *a*, schematic representation of the main steps of the screen. *b*, validation of primary hits from the *in vitro* kinase assay screen. 90 candidate peptides were purified and incubated with recombinant Fpk1. Reactions were loaded onto SDS-polyacrylamide gels, and resolved proteins were visualized with Coomassie Blue (*left panels*) and incorporation of radioactivity by phosphorimaging (*right panels*). *c*, ClustalX alignments of Ark1, Prk1, and Akl1 protein sequences. The *blue box* represents the conserved serine/threonine kinase domains, and the *dashed lines* delimit protein-specific insertions. *d*, Coomassie Blue–stained and autoradiogram images after *in vitro* kinase assays assessing the ability of Fpk1 to phosphorylate Akl1 variants as indicated. *e*, serial 10-fold dilutions of BY4741 cells containing an empty vector (*Ev* (−)) or vectors expressing *GAL1* promoter–driven GST-Akl1^WT^, GST-Akl1^AA^, or GST-Akl1^KD^ were spotted onto plates containing 2% raffinose (*Raff.*) or 2% galactose (*Gal.*). *f*, micrographs of rhodamine phalloidin–stained BY4741 cells containing either an empty vector (−) or vectors expressing *GAL1* promoter–driven GST-Akl1^WT^ or GST-Akl1^AA^. Galactose induction from raffinose cultures was for 2 h. Quantification of the extent of actin polarization is graphed as a representation of the normalized means ± S.D. *Error bars* represent S.D. Statistical significance was determined using Student's *t* test; *n.s.*, not significant; *p* > 0.05 (***) based on biological duplicates with *n* > 100 cells/replicate. *g*, fluid-phase endocytosis was also monitored in the cells used in *f*. Quantification of the extent of Lucifer yellow accumulation in the vacuole is graphed as a representation of the normalized means ± S.D. *Error bars* represent S.D. Statistical significance was determined using Student's *t* test; *p* < 0.0005 (***) based on biological duplicates with *n* > 100 cells/replicate. *DIC*, differential interference contrast; *O/E*, overexpression; *KD*, kinase-dead.

Previous studies have suggested that Fpk1 preferentially phosphorylates serine residues within the sequence R*X*S(L/Y)(D/E) (16). A search of the yeast proteome revealed that 90 peptides within 81 proteins match this consensus sequence (Table S2). DNA fragments encoding these 90 peptides (representing 15 amino acids in total length) were cloned and expressed as GST fusions in *Escherichia coli* (Fig. S2, a–c). Doping of crude bacterial lysates with purified Fpk1 and radioactive ATP yielded 36 potential direct substrates in 32 different proteins (Fig. 3*a* and Fig. S2d). These candidate substrates were then purified on GSH resin and retested; 32 peptides from 25 proteins were again found to be directly phosphorylated by Fpk1 (Fig. 3*b* and Fig. S2e). Among these were the known Fpk1 substrates Dnf1, Dnf2, and Ypk1 (16, 17), demonstrating the utility of this screen. Of the 22 putative novel Fpk1 protein substrates, only one is implicated in regulating endocytosis, Akl1.

Akl1 is a kinase related to Ark1 and Prk1, known regulators of endocytosis (28, 29). In addition, we had observed previously that Akl1 phosphorylation is altered upon TORC2 inhibition (15). Akl1, Ark1, and Prk1 have highly similar kinase domains in their N termini but divergent C termini (Fig. 3*c*). Our *in vitro* screen suggested that Fpk1 phosphorylates Akl1 on residues Ser-960 and Ser-1072. These residues are in the C-terminal domain of Akl1 and are not obviously conserved in either Ark1 or Prk1. Fpk1 phosphorylated full-length, kinase-dead Akl1^D181Y^ but not Akl1^D181Y/S960A/S1072A^, suggesting that these two amino acids are the only residues phosphorylated by Fpk1 (Fig. 3*d* and Fig. S2f). In similar assay conditions, we did not observe phosphorylation of Akl1 by Fpk2 (Fig. S3).

### Akl1 links TORC2 to endocytosis

Relative to Ark1 and Prk1, the functions of Akl1 are much less well-understood. Overexpression of Ark1 or Prk1 is toxic (24, 30), and so we asked whether the same is true of Akl1. Indeed, overexpression of WT Akl1 is toxic to cells, but overexpression of kinase-dead Akl1^D181Y^ does not affect growth (Fig. 3*e*). Overexpression of Akl1^S960A/S1072A^ that cannot be phosphorylated by Fpk1 was also toxic, suggesting that Fpk1-mediated phosphorylation is not required for Akl1 kinase activity. Actin polarization was not affected in cells overexpressing WT Akl1 or Akl1^S960A/S1072A^ (Fig. 3*f*), but LY uptake in these cells was significantly reduced (Fig. 3*g*), suggesting that, like Ark1 and Prk1, Akl1 also regulates endocytosis.

**Figure 4.**
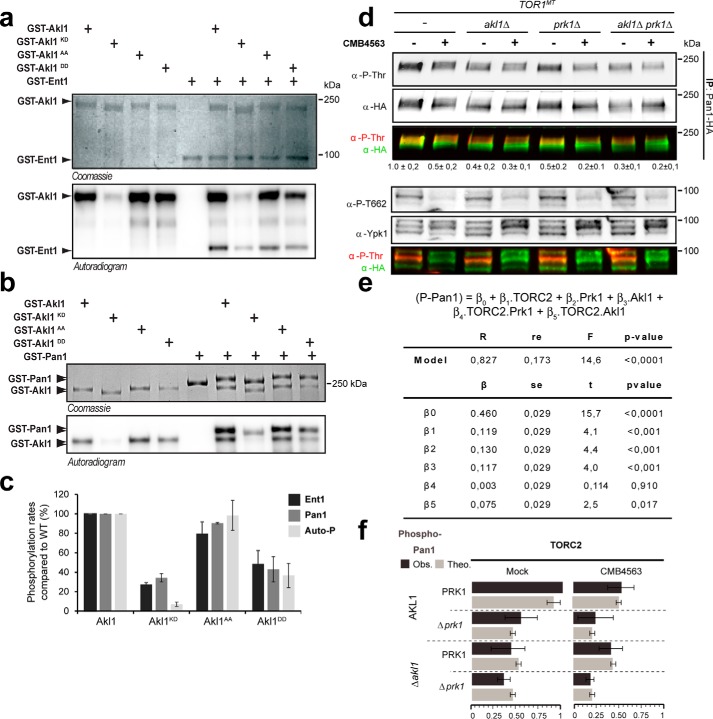
**By inhibiting Fpk1, TORC2/Ypk1 promotes Akl1 activity.** *a*, Coomassie Blue and autoradiogram SDS-PAGE images after *in vitro* kinase assays assessing the ability of the indicated Akl1 variants to phosphorylate Ent1. *b*, Coomassie Blue and autoradiogram SDS-PAGE images after *in vitro* kinase assays assessing the ability of the indicated Akl1 variants to phosphorylate Pan1. *c*, quantification (from *a* and *b*) of Ent1, Pan1, and autophosphorylation *(Auto-P*) by Akl1 variants. Plotted are normalized means ± S.D. based on biological triplicate experiments. *Error bars* represent S.D. *d*, Western blot assessing the extent of threonine phosphorylation in Pan1-5HA immunoprecipitated from cells of the indicated genotype treated or not with CMB4563 for 30 min. *e*, multiple linear regression assessment (IBM SPSS Statistics 24) of the contribution of TORC2, Prk1, Akl1, and combinations to Pan1 phosphorylation (*P-Pan1*) based on data from *d* in *TOR1^MT^* alone or combined with *akl1*Δ and/or *prk1*Δ. The analysis was performed with a data set collected from biological triplicates. The obtained model explains 82.7% of the total observed variance (*R*^2^, residual error (*re*) = 17.3%) and has a high statistical significance (Fisher's test (*F*), *p* < 10^−5^). The model shows that the contribution of TORC2, Akl1, and Prk1 are statistically significant (β1, β2, and β3; Student's *t* test, *p* < 0.001). In addition, the contribution of TORC2 via Akl1 (*TORC2.Akl1*), β3, is significant with *p* < 0.05, which is not the case for the contribution of TORC2 via Prk1 (*TORC2.Prk1*) with *p* > 0.5. *f*, simulation based on the model (theoretical model (*Theo.*), *light gray bars*; observed data (*Obs.*), *dark gray bars*). *Error bars* represent S.D. *IP*, immunoprecipitation; *KD*, kinase-dead; *Akl1^DD^*, Akl1 S960/1072D.

We tested whether Akl1, like Ark1 and Prk1, can phosphorylate endocytic coat proteins. We found that Akl1^WT^ and Akl1^S960A/S1072A^ efficiently phosphorylate Ent1 (Fig. 4, *a* and *c*) and Pan1 (Fig. 4, *b* and *c*) *in vitro*, whereas the activities of phosphomimetic Akl1^S960D/S1072D^ and kinase-dead Akl1^D181Y^ were obviously compromised (<50% activity of WT). This is consistent with the notion that Fpk1-mediated phosphorylation inhibits kinase activity of Akl1. We determined the relative contributions of Akl1 and Prk1 to Pan1 phosphorylation *in vivo* (Fig. 4*d*). Deletion of either *AKL1* or *PRK1* led to an ∼50% drop in anti-phosphothreonine signal on Pan1, suggesting that both kinases contribute to Pan1 phosphorylation in growing cells. Importantly, TORC2 inhibition also reduced Pan1 phosphorylation by ∼50% in WT and *prk1* cells but not in *akl1* or *alk1 prk1* cells. The data from multiple repetitions of these experiments were assessed using multiple linear regressions (Fig. 4, *e* and *f*). From this analysis, we calculated that Akl1 contributes 19.2% (β3 + β5 = 11.7 + 7.5%) of the observed variance in Pan1 phosphorylation and that this contribution is significantly linked to TORC2 activity (*p* value (β5) < 0.05). Prk1 contributes to 13% of this variance (β2), but this variance is not significantly associated with TORC2 activity (*p* value (β4) > 0.05). These data suggest that TORC2 regulates coat protein phosphorylation via a Ypk1–Fpk1–Akl1 kinase cascade.

**Figure 5.**
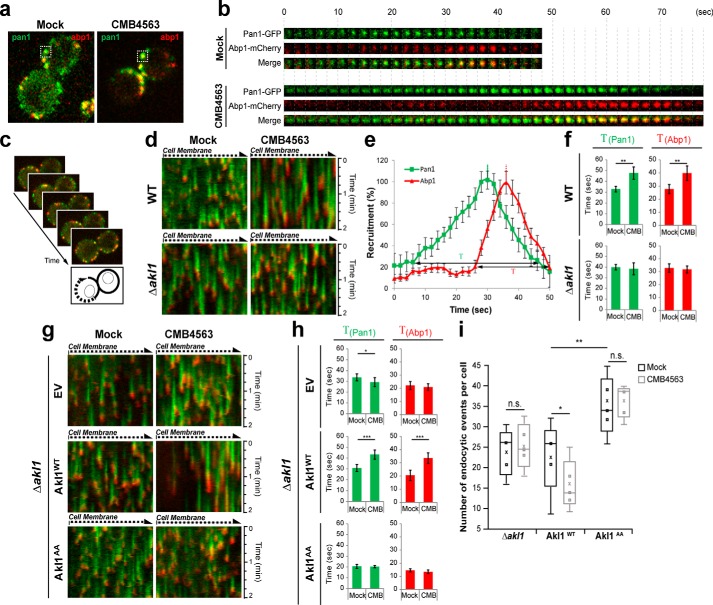
**Akl1 regulates assembly of the endocytic patch.** *a*, localization of Pan1-GFP and Abp1-mCherry in *TOR1^MT^* cells after 30-min mock (DMSO) or CMB4563 treatment. *b*, Pan1-GFP and Abp1-mCherry signals from an individual endocytic event (*boxed* in *a*) observed over time (one frame/2 s). *c*, illustration of how the kymographs in *d* were obtained by following a line along the plasma membrane around an entire cell. *d*, Pan1-GFP and Abp1-mCherry kymographs were generated from *TOR1^MT^* and *TOR1^MT^ akl1*Δ mutant strains treated or not with CMB4563. *e*, quantification of Pan1-GFP and Abp1-mCherry patch lifetimes assessed from a single, mock-treated cell. *f*, quantification of Pan1-GFP and Abp1-mCherry patch lifetimes determined from *TOR1^MT^* and *TOR1^MT^ akl1*Δ cells as assessed in *d*. Bar graphs show Pan1-GFP and Abp1-mCherry lifetime means ± S.D. *Error bars* represent S.D. Statistical parameters were extracted (Student's *t* test; **, *p* < 0.005) based on five patches/kymograph. Seven kymographs were analyzed. *g*, Pan1-GFP and Abp1-mCherry kymographs were generated from *TOR1^MT^ akl1*Δ cells expressing Akl1^WT^, Akl1^AA^, or an empty plasmid. *h*, Pan1-GFP and Abp1-mCherry patch lifetimes determined from the cells used in *g* and as described in *f. Error bars* represent S.D. *i*, box plots of the number of endocytic events determined from the cells used in *g*. The *squares* represent the number of endocytic events per kymograph. The *cross* represents the mean value. *Error bars* represent S.D. Statistical parameters were extracted (Student's *t* test; *n.s.*, not significant; *, *p* < 0.05; **, *p* < 0.005). *EV*, empty vector; *CMB*, CMB4563.

Finally, we assessed in more detail the role of Akl1 phosphorylation in the regulation of endocytosis downstream of TORC2. To monitor endocytosis kinetics, we followed the recruitment and residency times of the coat protein Pan1 (tagged with GFP) and the actin-binding protein Abp1 (tagged with mCherry) to endocytic patches at the plasma membrane (Fig. 5*a*) (29). Consistent with previous results (31, 32), in mock-treated cells Pan1 was recruited to an endocytic patch and resided there for ∼30 s (Fig. 5*b*). Abp1 recruitment began with an ∼20-s delay and associated with Pan1 for ∼10 s after which time the patch budded into the cytoplasm, representing completion of a successful endocytic event. Inhibition of TORC2 greatly extended the residency times of both Pan1 and Abp1, and endocytic patches often failed to resolve/bud inward (Fig. 5*b*).

To better quantify this affect we followed, as a kymograph (Fig. 5*c*), multiple endocytic events over time (Fig. 5, *d* and *e*). We observed that inhibition of TORC2, but not TORC1, indeed increased the residency times of both Pan1 and Abp1 by ∼50% (Fig. 5*f* and Fig. S4). Importantly, this increase was not observed in *akl1* cells. To probe this effect further, we retested *TOR1^MT^ akl1* cells transformed with an empty plasmid (no insert) or encoding WT Akl1 or Akl1 that cannot be phosphorylated by Fpk1 (Fig. 5, *g* and *h*). Again, we found that inhibition of TORC2 increased the residency times of both Pan1 and Abp1 in the presence, but not absence, of Akl1. Strikingly, in mock-treated cells expressing Akl1^S960A/S1072A^, the residency times of both Pan1 and Abp1 were already reduced by ∼50%, and this short residency time was unaltered by TORC2 inhibition. Furthermore, we observed that TORC2 signaling affects the number of endocytic events per cell in an Akl1-dependent manner (Fig. 5*i*). Specifically, inhibition of TORC2 reduces the number of endocytic membrane patches in cells expressing WT Akl1 but not in *akl1* cells; cells expressing Akl1^S960A/S1072A^ presented significantly more endocytic events, and this number was again unaffected by TORC2 inhibition. Collectively, these results demonstrate the existence of a TORC2–Ypk1–Fpk1–Akl1 kinase cascade that regulates coat protein phosphorylation and endocytosis dynamics.

## Discussion

**Figure 6.**
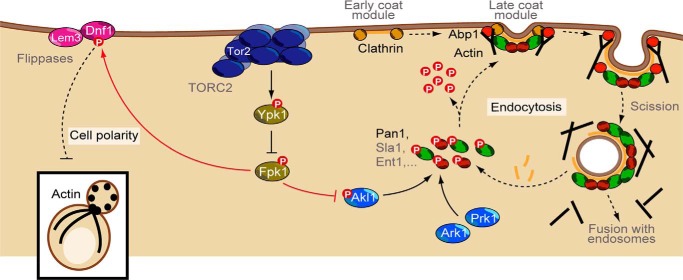
**Model illustrating that TORC2 regulates endocytosis via Akl1 and cell polarity via aminophospholipid flippases.** *Solid lines* highlight direct phosphorylation events. *Arrows* denote activation, and *bars* denote inhibition.

Depletion of TORC2 activity in yeast cells has long been known to lead to the loss of actin polarization as well as an eventual cessation of clathrin-mediated endocytosis (9, 13, 33). It has been assumed that the regulation of these two distal effectors of TORC2 is mechanistically coupled. However, using a novel potent TOR inhibitor, CMB4563, we have demonstrated that signaling to these effectors occurs via separate pathways. Specifically, we found that TORC2-dependent signaling bifurcates at the level of the flippase kinase Fpk1 with signaling to the actin polarization machinery being mediated in large part via amino phospholipid flippases. Starting with an *in silico* selection followed by an *in vitro* screen, we found that signaling to the endocytic machinery is mediated in large part via the Ark/Prk-related kinase Akl1 (Fig. 6). TORC2/Ypk1 plays an important role in the homeostatic regulation of membrane tension (15, 34, 46). Thus, it makes sense that part of this regulation would include control of endocytosis, a process that inherently impacts membrane tension. TOR signaling is robustly conserved from yeast to human (35). In light of this, it will also be interesting to determine whether the mammalian orthologs of Ark1/Prk1/Akl1, GAK1 and AAK1 (28), also regulate clathrin-mediated endocytosis downstream of mTORC2 in mammalian cells.

Acute inactivation of TORC2 with CMB4563 occurs within a minute (Fig. S1c) as evidenced by loss of phosphorylation on the TORC2 target site Thr-662 in Ypk1. Ypk1 activity is consequently reduced; it no longer phosphorylates/inhibits Fpk1 (16). As shown here, Fpk1 phosphorylates and thus hyperactivates Akl1 on residues Ser-960 and Ser-1072 to inhibit its kinase activity. These results are consistent with recent work from Thorner and co-workers (13); however, we have extended this previous work by dissecting the consequences that dysregulation of TORC2-mediated coat protein phosphorylation has on the kinetics of endocytosis.

Hypophosphorylated endocytic coat proteins are assembled into nascent endocytic patches during the initial steps of endocytosis. Later, when an endocytic vesicle has been pinched off from the plasma membrane, coat proteins are phosphorylated, facilitating their disassembly (25). We show here that TORC2 inhibition blocks endocytosis (Fig. 2*d*). Upon further examination, we observed that under these conditions there is a dramatic extension in the lifetime of Pan1 and Abp1 at the endocytic patch (Fig. 5*d*). Importantly, in *akl1*Δ or *AKL1^AA^* cells, TORC2 signaling to the endocytic patches is disrupted as evidenced by the observation that CMB4563 treatment no longer increases Pan1 or Abp1 patch lifetimes in these cells. Together, these data are consistent with TORC2 regulating endocytosis dynamics via regulation of Akl1 activity. When Akl1 activity is inhibited, for example upon TORC2 inhibition, an abundance of hypophosphorylated coat proteins at the endocytic site might promote incorporation of coat proteins, explaining aberrantly long-lived patches. A gradual gain of hypophosphorylated coat proteins could explain why it takes an otherwise unusually long period of time (∼30 min) to observe a delay of endocytosis upon acute TORC2 inhibition. Following this logic, an excess of hyperphosphorylated coat proteins, as might be anticipated in cells expressing Akl1^AA^, would be predicted to antagonize coat protein incorporation and lead to short-lived endocytic patches, which is what we observed (Fig. 5*h*). Alternatively, or additionally, TORC2 may control other pathways apart from that of Akl1-mediated coat protein phosphorylation to regulate endocytosis.

Our screen aiming for novel Fpk1 effectors yielded known targets of this kinase, Dnf1, Dnf2, and Ypk1 (16), confirming the validity of our approach. However, in addition to these known phosphosites, we also found novel phosphosites in Dnf1 (Ser-365) and Dnf2 (Ser-386 and Ser-403). The identification of these sites will help reveal, in future studies, how Fpk1-mediated phosphorylation of these aminophospholipid flippases regulates their ability to flip phosphatidylethanolamine to regulate, in turn, polarization of Cdc42 (36) and Rho1 (37).

Finally, the hits from our screen suggest additional, unanticipated targets of TORC2–Ypk1–Fpk1 signaling. These potential distal targets include lipid flipping at the *trans*-Golgi network via Dnf3 (38), cortical endoplasmic reticulum–plasma membrane tethering via the tricalbin Tcb3 (39), sphingolipid recycling via the Golgi-associated retrograde protein (GARP) complex subunit Vps54 (40), and autophagy via Atg13 in addition to other pathways (41–43). Lastly, poorly understood targets such as the putative AAA-ATPase Yta6 and the kelch-like domain–containing protein Mds3 suggest that completely new targets of TORC2 signaling remain to be discovered.

## Materials and methods

### Strains and growth conditions

The yeast strains used in this study are listed in Table 1. Yeasts were grown in complete synthetic medium (CSM) buffered at pH 6.25 (Soerensen buffer) lacking the appropriate amino acid(s) to maintain plasmid selection when necessary and with the appropriate carbon source (2% glucose, raffinose, or galactose). Treatment with CMB4563, diluted in DMSO, at the indicated concentrations was performed for 12–30 min in exponentially growing cultures (44).

**Table 1:** **Yeasts strains used in this study**

Strain	Genotype	Source or Ref.
TB50	MATa *leu* 2–3,112 *ura* 3–52 *trp1 his* 3 *rme* 1 HMLa	Lab stock
MS184	TB50a 3HA-*TOR1M2282T*	15
MS185	TB50a 3HA-TOR1WT *TOR2M2286T*	lab stock
MS186	TB50a 3HA-*TOR1M2282T TOR2M2286T*	15
SE14-20	TB50a 3HA-*TOR1M2282T fpk1::NatMX*	15
SE15-28	BY4741 pRS426-Gal1-GST-His_6_-TEV-AKL1	Lab stock
DRY87	TB50a 3HA-*TOR1M2282T lem3::HphMX4*	This study
DRY121	TB50α 3HA-*TOR1M2282T* Abp1-mcherry[HphMX4]	This study
DRY270	TB50a 3HA-*TOR1M2282T akl1::KanMX6*	This study
DRY304	TB50a 3HA-*TOR1M2282T prk1::NatMX4*	This study
CB3162	TB50a 3HA-*TOR1M2282T akl1::KanMX6 prk1::NatMX4*	This study
CB3185	TB50α 3HA-*TOR1M2282T* Abp1-mcherry[HphMX4] *akl1::KanMX6*	This study
CB3197	TB50a 3HA-*TOR1M2282T akl1::KanMX6* Abp1-mcherry::[HphMX4] pRS414-PAN1-GFP-TADH1	This study
BY4741	MATa *his3* Δ1 *leu* 2Δ0 met15Δ0 *ura* 3Δ0	Lab stock

### Plasmid and strain constructions

Plasmid constructs, other than those used in the Fpk1-target screen, are shown in Table 2. For the screen of Fpk1 peptide substrates, a library of plasmids composed of the 90 encoded candidate peptides was constructed by recombination of the linearized pST0 vector generated by PCR (forward primer, GGATATCGGGGATCCGAATTCTG; reverse primer, CATGGAGCCCTGAAAATAAAGATTCTC) with a 1:1 molar ratio of primer dimers (Table S1, Fig. S2, b–d, and Fig. 1, *a* and *b*) using the strategy of Gibson *et al*. (45).

**Table 2:** **Plasmids used in this study** aa, amino acids.

Name	Description	Source or Ref.
pST0	pET42 GST-His_6_-TEV cleavage site	Lab stock
pCB1221	pST0-Dnf1 (aa 1404–1571)	This study
pCB1208	pST0-Orm1 (aa 1–85)	This study
pCB1558	pRS426-Gal1-GST-His_6_-TEV-AKL1 D181Y	This study
pCB1557	pRS426-Gal1-GST-His_6_-TEV-AKL1 S960A/S1072A	This study
pCB1567	pRS426-Gal1-GST-His_6_-TEV-AKL1 S960D/S1072D	This study
pSE14-03	pRS426-Gal1-GST-His_6_-TEV-ENT1	Lab stock
pCB1574	pRS426-Gal1-GST-His_6_-TEV-PAN1	This study
pMS080	pRS415-ENT1-5HA-TADH1	Lab stock
pRS414	pBluescript II SK+, TRP1, CEN6, ARSH4	Lab stock
pMS082	pRS415-PAN1-5HA-TADH1	Lab stock
pMS123	pRS415-PAN1-GFP-TADH1	Lab stock
pMS120	pRS415-AKL1-5HA-TADH1	Lab stock
pCB1483	pRS415-AKL1-5HA-TADH1 S960A/S1072A	This study

### Immunoblotting assays

Yeast cells were grown to an *A*_600_ of 0.6–0.8 before the indicated treatment. Protein extracts and immunoprecipitations were performed as in Rispal *et al.* (15). Western blot membranes were analyzed and quantified using the Odyssey® IR imaging system (LI-COR Biosciences). Antibodies used include rabbit polyclonal anti-Sch9 (homemade; 1:5000), mouse monoclonal anti-phospho-Sch9^Ser-758^ (homemade; 1:2500), polyclonal goat anti-Ypk1 (Cell Signaling Technology; 1:1000); monoclonal mouse anti-phospho-Ypk1^Thr-662^ (homemade; 1:500); monoclonal mouse anti-HA (Sigma; 1:20,000), polyclonal rabbit anti-phosphothreonine (Life Technologies; 1:1000), and the appropriate fluorescent dye–coupled secondary antibodies (all Alexa Fluor–conjugated secondary antibodies, LI-COR Biosciences).

### Protein inductions and purifications

GST-Akl1, -Fpk1, -Prk1, -Ypk1, -Ent1, and -Pan1 fusion proteins were expressed from multicopy plasmids under the control of *GAL1* promoter in BY4741 cells. Precultures were grown overnight in CSM with 2% raffinose and freshly diluted in the same medium until an *A*_600_ of 0.8. Galactose (2%) was subsequently added to the culture, and the induction was done for 2 or 4 h depending on the experiment. GST-Dnf1, GST-Orm1, and the library of GST-tagged Fpk1candidate substrates were purified from *E. coli*. The corresponding plasmids were transformed and expressed in BL21*. Overnight cultures grown in Terrific Broth medium complemented with 50 μg/ml kanamycin were diluted to an *A*_600_ of 0.1, 0.1 mm isopropyl 1-thio-β-d-galactopyranoside was added at an *A*_600_ of 0.6–0.8, and then cultures were grown overnight at 18 °C.

Yeast/bacterial cell pellets were resuspended in PBS lysis buffer (10 ml/g) containing 10% glycerol, 5 mm CHAPS, Complete protease inhibitor mixture (Roche Applied Science; 1.5 tablets/50 ml), 1 mm PMSF (with 10 μg/ml lysozyme and 20 μg/ml DNase for bacterial pellets). Cells were mechanically broken using a French press. Following GSH-affinity chromatography and in some cases removal of the GST tag by TEV protease cleavage, purified proteins were dialyzed and/or loaded onto Superdex 200 10/30 (GE Healthcare) gel filtration column. The final buffer composition was 25 mm HEPES, pH 7.5, 150 mm KCl, 5 mm MgCl_2,_ 1 mm DTT, 2.5 mm CHAPS, and for purified kinases 50% glycerol.

### In vitro protein kinase assays and protein kinase–based peptide substrate screen

Cell pellets from 5-ml cultures of bacteria expressing GST-peptides were lysed with 250 μl of Bugbuster protein extraction reagent (Novagen) supplemented with Complete protease inhibitor mixture, and protein concentrations were normalized. Protein kinase assays were performed in 15 μl of kinase assay buffer (25 mm K-HEPES, 2.5 mm CHAPS, 5 mm MgCl_2_, 1 mm DTT, and 300 μm ATP including 2 μCi of [γ-^32^P]ATP) using 0.25 μm purified Fpk1 mixed with 0.5 μm purified substrates or 5 μl of total bacterial protein extract. Reactions were incubated for 30 min at 30 °C and stopped by addition of SDS-PAGE sample buffer. Samples were resolved by 7.5 or 10% SDS-PAGE, stained with Coomassie, dried, and analyzed with a Bio-Rad Molecular Imager.

### Endocytosis and actin assays

Microscopy experiments, as well as rhodamine phalloidin and Lucifer yellow stainings were performed as described (15). Confocal Z-stack images were acquired with a Zeiss LSM700 at 63× magnification.

### Microfluidics imaging

Strains expressing Pan1-GFP and Abp1-mCherry were grown in appropriate synthetic media overnight and diluted to an *A*_600_ of 0.1. When cells reached an *A*_600_ of 0.4–0.6, 100 μl were pipetted into flow chambers (sticky-Slide VI 0.4, Ibidi), fixed to a concanavalin A (0.5 μg/μl)–coated coverslip, incubated for 10 min, and washed once with fresh medium. The flow chamber was subsequently filled with CSM containing the appropriate treatment. Single-plane images were taken every 10 s for 2 min with a Nikon spinning-disc microscope (Eclipse C1) at 100× magnification. Collected images were treated and analyzed with Fiji ImageJ. Specifically, a six-pixel-thick curve was drawn along the plasma membrane of each selected cell. The average signals along this line were measured at each time point and fused in a final kymograph displaying both red and green channels. Endocytic events were counted, and their intensity and lifetime were measured.

## Author contributions

R. L., C. B., and M. P. formal analysis; R. L. funding acquisition; R. L., C. B., and M. P. writing-original draft; R. L. project administration; R. L., C. B., and S. B. H. writing-review and editing; C. B., D. R., and M. P. conceptualization; C. B., D. R., M. B., and M. P. investigation; C. B., D. R., and M. P. methodology; I. F. and S. B. H. resources; M. P. supervision.

## Supplementary Material

Supporting Information

## Footnotes

3 The abbreviations used are:

TOR: target of rapamycin
TORC: target of rapamycin complex
Fpk: flippase kinase
*TOR1^MT^*: *TOR1^M2282T^*
*TOR2^MT^*: *TOR2^M2286T^*
*Akl1^AA^*: Akl1 S960/1072A
LY: Lucifer yellow
GST: glutathione *S*-transferase
TEV: tobacco etch virus
CSM: complete synthetic medium
HA: hemagglutinin.
