# Supplementary material for: Target of rapamycin complex 2–dependent phosphorylation of the coat protein Pan1 by Akl1 controls endocytosis dynamics in Saccharomyces cerevisiae
Source: J Biol Chem. 2018 Jun 12;293(31):12043–53. doi: 10.1074/jbc.RA117.001615 (PMC6078453; doi:10.1074/jbc.RA117.001615)
Supplement: Supporting Information [file supp_293_31_12043__index.html]

Target Of Rapamycin Complex 2–dependent phosphorylation of the coat protein Pan1 by Akl1 controls endocytosis dynamics in Saccharomyces cerevisiae — TORC2 regulates endocytosis via Akl1. — Target of rapamycin complex 2–dependent phosphorylation of the coat protein Pan1 by Akl1 controls endocytosis dynamics in Saccharomyces cerevisiae — TORC2 regulates endocytosis via Akl1 — Supporting Information 

# Target of rapamycin complex 2–dependent phosphorylation of the coat protein Pan1 by Akl1 controls endocytosis dynamics in *Saccharomyces cerevisiae*

## Supporting Information

- Supporting Information - 4 supporting figures, 2 supporting tables
