# Supplementary material for: Target of rapamycin complex 2–dependent phosphorylation of the coat protein Pan1 by Akl1 controls endocytosis dynamics in Saccharomyces cerevisiae
Source: J Biol Chem. 2018 Jun 12;293(31):12043–53. doi: 10.1074/jbc.RA117.001615 (PMC6078453; doi:10.1074/jbc.RA117.001615)
Supplement: Supporting Information [file supp_RA117.001615_134887_2_supp_150685_pv83gr.pdf]

SUPPORTING INFORMATION

Supplemental figure 1

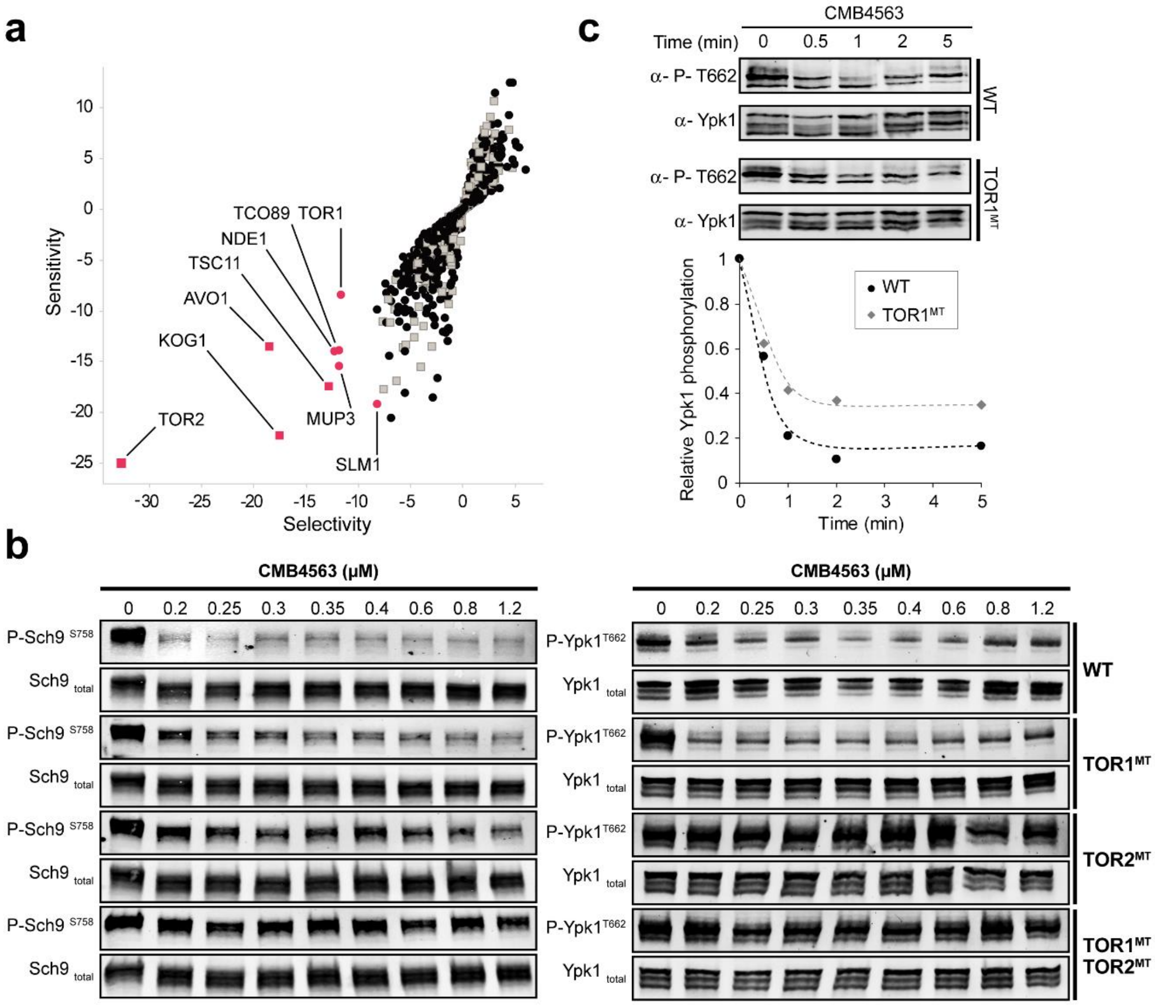

Supplemental figure 1: CMB4563 is a potent and fast inhibitor of TOR1 and TORC2. a, Heterozygous single deletion mutant strains covering 6200 genes (Open Biosystems) were grown as a pool in the presence or absence of 0.5 µM CMB4563 for 20 generations. The relative abundances of the individual strains were then compared. Sensitivity of individual deletion mutants are observed on the vertical axis and associated with a selectivity on the horizontal axis which was determined by statistical analysis to correct for how frequently particular mutants scored in multiple assays. The most selective and sensitive strains are found in the bottom left corner (1). **b,** Western blots assessing the respective activity of TORC1 and TORC2 after 12-minute CMB4563 treatments to cells of the indicated genotypes. The corresponding merge channels version is in Fig. 1d. TORC1 activity is followed by Sch9$^{P\text{-}S758}$ and TORC2 by Ypk1$^{P\text{-}T662}$ phosphorylation. **c,** Western blot and corresponding quantification assessing the kinetics of Ypk1^{T662} dephosphorylation in *TOR1^{WT}* and *TOR1^{MT}* strains with 0.8 µM CMB4563 treatment at indicated time points (above, minutes).

Supplemental figure 2

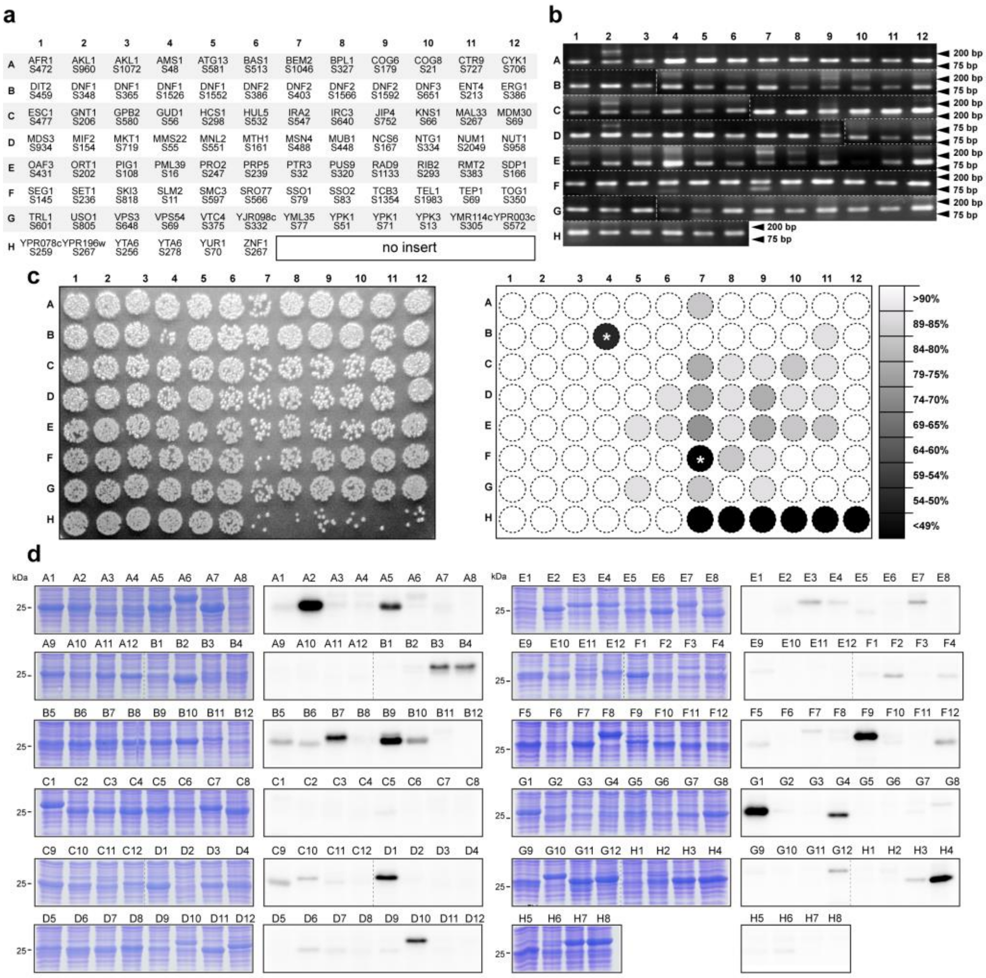

e

Protein	Function	Size (aa)	Motif	Putative Phospho-S Position (aa)	Detected S-Phospho
AKL1	Endocytosis	1108	ssvstrqpRqSLDlnfqevnl	960	YES
			mrksfaraRqSLDlervrrea	1072	YES
ATG13	Vesicles traficking/Autophagy/TOR pathway factor	738	stsafnsrRnSLDkssnkqgm	581	YES
DNF1	Lipid transport	1571	*ktvgtsgpRsSLDsidsyrvs*	*348*	*NO*
			yrvsadygRpSLDydnleqga	365	YES
			grgpstnvRsSLDrtreqmia	1526	YES
			nrysveraRtSLDlpgvtnaa	1552	YES
DNF2	Lipid transport	1612	rnsrsfgpRgSLDsigsyrms	386	YES
			yrmsadfgRpSLDyenlnqtm	403	YES
			asargnklRtSLDrtreemla	1566	YES
DNF3	Lipid transport	1656	dapiekghRpSLDnfpksrns	651	YES
HCS1	DNA synthesis	683	*arlldsnkRhSLDilskknti*	*298*	*NO*
KNS1	TOR pathway factor	737	*nsfltdnlRnSLDlnqadnpl*	*66*	*NO*
MAL33	Maltose metabolism	468	*qpefvtdpRlSLDsffemirv*	*267*	*NO*
MDS3	TOR pathway factor	1487	enafsatpRaSLDgqmlgksl	934	YES
MTH1	Glucose sensing	433	*fpnhklitRvSLDealpktfy*	*161*	*NO*
MSN4	Transcription regulation	630	itptpafqRrSYDismnssfk	488	YES
NCS6	tRNA modification	359	*ctycgvfrRqSLDrgaaklgi*	*167*	*NO*
NTG1	DNA repair	399	*qflppddpRsSLDwdlqsqly*	*334*	*NO*
PTR3	Amino acids sensing	678	*ehdlylpqRlSLDgatgtdes*	*32*	*NO*
RAD9	DNA repair	1309	*llpsgesfRlSLDspskqgii*	*1133*	*NO*
SEG1	Eisosome	960	*rqkskthqRiSYDeaqrtfkd*	*145*	*NO*
SET1	Chromatin	1080	*rnslillpRiSYDryslgppp*	*236*	*NO*
SLM2	Membrane stress	656	msyqrnsaRaSLDlrsqyqql	11	YES
SSO1	Vesicles traficking	290	neeqashlRhSLDnfvaqatd	79	YES
SSO2	Vesicles traficking	295	*seeqemelRrSLDdyisqatd*	*83*	*NO*
TCB3	PI4P regulation	1545	lnstsvtpRaSLDydpsvpnt	1354	YES
TOG1	Fatty acid metabolism	794	*griistmsRrSLDaglnrwey*	*350*	*NO*
			snssvvpvRvSYDassststv	71	YES
YPR003c	Unkown function	754	*ddymmnmkRnSLDvegteeie*	*572*	*NO*
YTA6	Assymetric protein	754	erktnyksRaSLDnfssssks	255	YES
			kidnslikRrSLDvvrtsger	278	YES

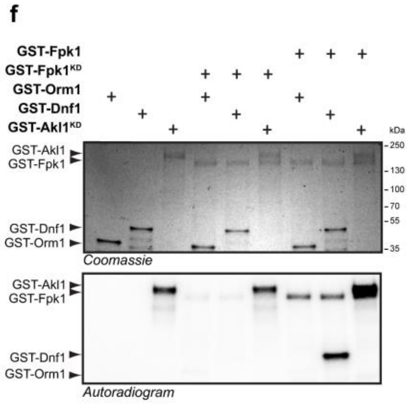

Supplemental figure 2: **Fpk1 substrate screen. a**, Organization of the 96-well-plate containing negative controls and the primer dimers corresponding to the 90 candidate peptide substrates. **b**, PCR products of the primer dimers analysed on 2% agarose gels **c,** Left Panel: Gibson assembly reactions were transformed into bacteria. 1/10 of the transformed cells were spotted onto LB plates containing kanamycin, including multiple negative controls corresponding to linearized vector without insert. Right Panel: Heat map reporting the cloning efficiency assessed with respect to the negative controls. The (*) indicated failed wells in which the cloning/transformation initially failed (these were subsequently repeated, successfully). **d**, Results of the initial *in vitro* kinase assays screen. Dashed lines define splicing of the gels. Bacterial lysates containing one of the 90 candidate peptides were individually incubated with GST-Fpk1 and ^{32}P-ATP for 30 min at 30°C. Reactants were subsequently resolved by SDS-PAGE, analysed by Coomassie stain (left panel) and autoradiography (right panel). **e**, Table recapitulating the peptides identified in both the initial and secondary screens (left) **f**, *In vitro* kinase assay assessing the ability of GST-Fpk1$^{WT/KD}$ to phosphorylate GST-Akl1^{KD}, GST-Orm1 (aa 1-85; negative control) or GST-Dnf1 (aa 1404-1571; positive control).

Supplemental figure 3:

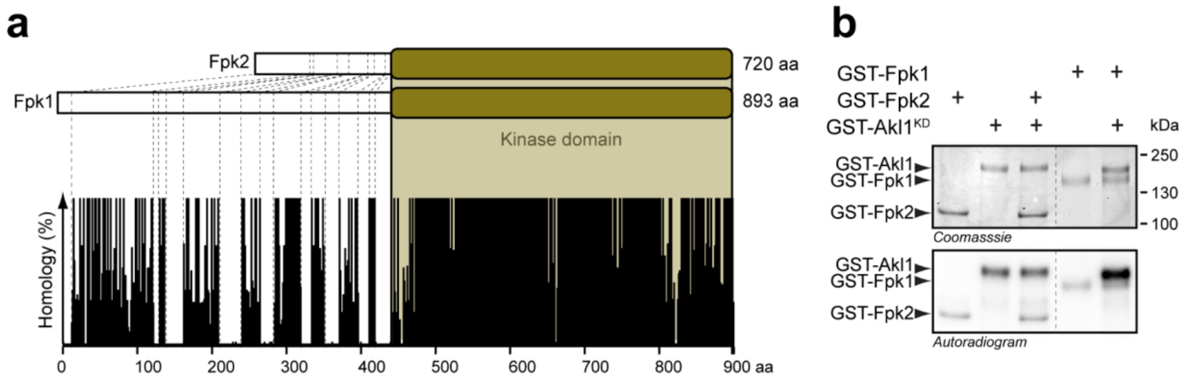

Supplemental figure 3: Akl1 is not a target of Fpk2 *in vitro*. a, ClustalX alignments of Fpk1 and Fpk2 protein sequences. The green box represents the conserved kinase domain and the dashed lines delimit sequence insertions. **b,** Coomassie blue and autoradiogram SDS-PAGE images after *in vitro* kinase assay assessing the ability of Fpk1 and Fpk2 to phosphorylate Akl1. Dashed lines define splicing of the gel.

Supplemental figure 4

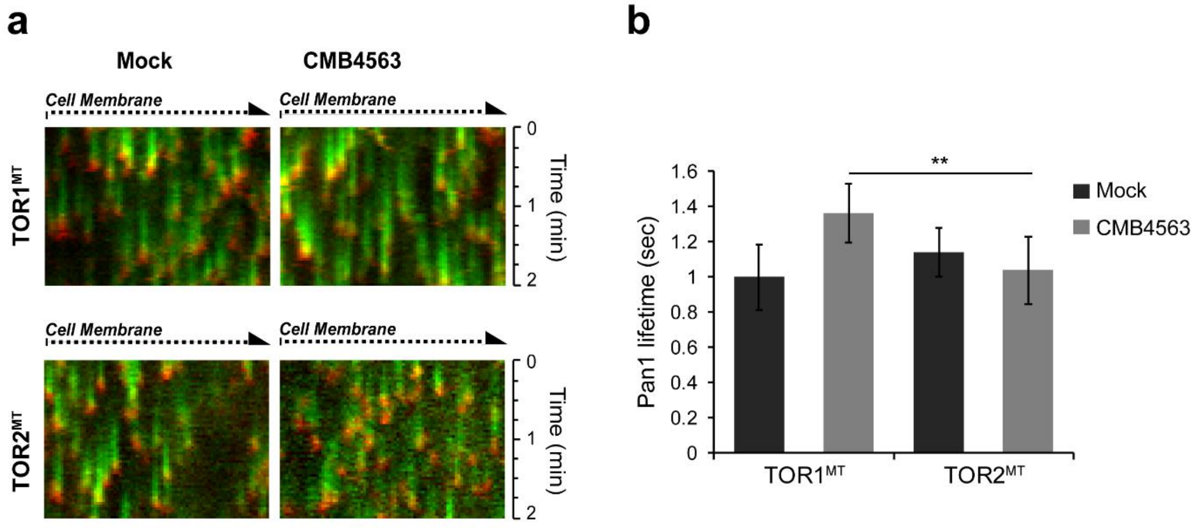

Supplemental figure 4: The inhibition of TORC1 does not cause delay the endocytosis process. a, Pan1-GFP and Abp1-mCherry kymographs were generated from *TOR1^{MT}* or *TOR2^{MT}* strains treated or not with CMB4563 for 30 min. **b**, Quantification of Pan1-GFP and Abp1-mCherry patch lifetimes determined from *TOR1^{MT}* or *TOR2^{MT}* cells as assessed in a. Bar graphs of Pan1-GFP lifetimes means, +/- standard deviation. Statistical parameters were extracted (Student's test: $P < 0.005$(**)) based on five patches/kymograph. Five kymographs were analysed.

Supplemental table 1

a

Forward primers

	1	2	3	4	5	6
A	TTTATTTTCAGGGCTCCATGCAGCCGCCGCCGCGCAGCAGCAAACGCCCGAGCCTGGATG	TTTATTTTCAGGGCTCCATGAGCAGCGTGAGCACCCGCCAGCCGCGCCAGAGCCTGGATCTGA	TTTATTTTCAGGGCTCCATGATGCGCAAAAGCTTCGCGCGCGCGCGCCAGAGCCTGGATCTGGA	TTTATTTTCAGGGCTCCATGCTGCCGAAATTCTACGATAAAAAACGCATTAGCCTGGATCATG	TTTATTTTCAGGGCTCCATGAGCACCAGCGCGTTCAACAGCCGCCGCAACAGCCTGGATA	TTTATTTTCAGGGCTCCATGAGCGATACCTTCAACCCGGAATACCGCACCAGCCTGGATA
B	TTTATTTTCAGGGCTCCATGCTGGCGGAAATGCTGAAACAGTTCCGCTGGAGCCTGGATC	TTTATTTTCAGGGCTCCATGAAAACCGTGGGCACCAGCGGCCCGCGCAGCAGCCTGGATAGCA	TTTATTTTCAGGGCTCCATGTACCGCGTGAGCGCGGATTACGGCCGCCCGAGCCTGGATTACGA	TTTATTTTCAGGGCTCCATGGGCCGCGGCCCGAGCACCAACGTGCGCAGCA	TTTATTTTCAGGGCTCCATGAACCGCTACAGCGTGGAACGCGCGCGCACCAGCCTGGATCTGCCGGGCGTGAC	TTTATTTTCAGGGCTCCATGCGCAACAGCCGCAGCTTCGGCCCGCGCGGCAGCCTGGATAGCAT
C	TTTATTTTCAGGGCTCCATGGAACCGGAAAAAGATGATATTATTCGCAGCAGCCTGGATA	TTTATTTTCAGGGCTCCATGTACCTGGATAACGATGCGATTCTGCGCAGCAGCCTGGAT	TTTATTTTCAGGGCTCCATGGTGCATGGCACCAGCCTGATGGATCGCAGCAGCCTGGATA	TTTATTTTCAGGGCTCCATGAACGGCATTATTCGCTTCGTGAACCGCAACAGCCTGGATC	TTTATTTTCAGGGCTCCATGGCGCGCCTGCTGGATAGCAACAAACGCCATAGCCTGGAT	TTTATTTTCAGGGCTCCATGATGTTCATTGCGCTGGATAAAAAACGCCTGAGCCTGGATG
D	TTTATTTTCAGGGCTCCATGGAAAACGCGTTCAGCGCGACCCCGCGCGCGAGCCTGGATGGCCAGAT	TTTATTTTCAGGGCTCCATGACCTACAAACGCAAATACAGCACCCGCTACAGCCTGGATA	TTTATTTTCAGGGCTCCATGAGCCTGACCAGCGGCGAATTCAACCGCCTGAGCCTGGATA	TTTATTTTCAGGGCTCCATGCGCAGCCAGAAAATTGTGACCGAACGCCTGAGCCTGGATA	TTTATTTTCAGGGCTCCATGACCAACGAACTGGATGTGACCATTCGCAAAAGCTACGATGTGAGCCTG	TTTATTTTCAGGGCTCCATGTTCCCGAACCATAAACTGATTACCCGCGTGAGCCTGGAT
E	TTTATTTTCAGGGCTCCATGAGCTTCAAAATTAACCGCCATACCCGCCCGAGCTACGATA	TTTATTTTCAGGGCTCCATGATTGTGAAAAAAAGCCTGAAAGATCGCCATAGCCTGGATG	TTTATTTTCAGGGCTCCATGCCGTTCATTGGCGGCGAAGATCATCGCTACAGCCTGGATA	TTTATTTTCAGGGCTCCATGCTGGAAGTGCGCCTGAAAAGCATTCGCCATAGCCTGGAT	TTTATTTTCAGGGCTCCATGGATGCGGATCTGATTAAAGCGAAACGCATTAGCCTGGATGC	TTTATTTTCAGGGCTCCATGAGCGAAATGGAAGTGGAAGAACTGCGCCTGAGCCTGGATA
F	TTTATTTTCAGGGCTCCATGCGCCAGAAAAGCAAAACCCATCAGCGCATTAGCTACGATG	TTTATTTTCAGGGCTCCATGCGCAACAGCCTGATTCTGCTGCCGCGCATTAGCTACGATC	TTTATTTTCAGGGCTCCATGATTGGCCTGGTGGAAGTGCTGATGCGCTGCAGCCTGGATC	TTTATTTTCAGGGCTCCATGATGAGCTACCAGCGCAACAGCGCGCGCGCGAGCCTGGATCTGCGCAGCCAGT	TTTATTTTCAGGGCTCCATGCGCGTGACCTTCATTCCGCTGAACCGCCTGAGCCTGGATA	TTTATTTTCAGGGCTCCATGGATGCGCTGCAGCTGAAATTCAGCCGCTTCAGCCTGGATG
G	TTTATTTTCAGGGCTCCATGGATGATGAAATTGAAACCGCGTTCCGCCGCAGCCTGGATT	TTTATTTTCAGGGCTCCATGCTGGAAACCGAACTGAAAAACGTGCGCGATAGCCTGGATG	TTTATTTTCAGGGCTCCATGACCATTACCGTGGATATGTTCGAACGCGAAAGCTACGATG	TTTATTTTCAGGGCTCCATGACCCCGCGCGCGCGCCGCAGCAGCCGCCTGAGCCTGGATA	TTTATTTTCAGGGCTCCATGCAGCTGCCGGGCGATGCGCGCGTGCGCATTAGCCTGGATA	TTTATTTTCAGGGCTCCATGGTGCAGCAGTTCGGCTACGATTGGCGCCTGAGCCTGGATA
H	TTTATTTTCAGGGCTCCATGACCCTGCTGAAATACCGCTACGTGCGCACCAGCCTGGATA	TTTATTTTCAGGGCTCCATGCAGCCGGAAATTGTGACCGATAGCCGCCTGAGCCTGGATA	TTTATTTTCAGGGCTCCATGGAACGCAAAACCAACTACAAAAGCCGCGCGAGCCTGGATAACT	TTTATTTTCAGGGCTCCATGAAAATTGATAACAGCCTGATTAAACGCCGCAGCCTGGATG	TTTATTTTCAGGGCTCCATGTACTTCCCGAAAGTGAAACTGAGCCGCAACAGCTACGATG	TTTATTTTCAGGGCTCCATGCAGCCGGAATTCGTGACCGATCCGCGCCTGAGCCTGGATAGCT

	7	8	9	10	11	12
A	TTTATTTTCAGGGCTCCATGCCGGCGACCCCGAACCAGAAAATGCGCGATAGCCTGGATA	TTTATTTTCAGGGCTCCATGAGCTTCCAGTTCTACCGCGGCTACCGCGCGAGCTACGATG	TTTATTTTCAGGGCTCCATGAAAGTGCTGGAAGAAAACAAAGAACGCCTGAGCCTGGATG	TTTATTTTCAGGGCTCCATGGATGATCTGACCGAAGAACAGAAACGCCTGAGCCTGGATT	TTTATTTTCAGGGCTCCATGGCGCTGGAAATTCTGCGCAAAGTGCGCGATAGCCTGGATA	TTTATTTTCAGGGCTCCATGGCGTTCAGCCGCGGCGCGCTGGTGCGCGAAAGCCTGGATC
B	TTTATTTTCAGGGCTCCATGTACCGCATGAGCGCGGATTTCGGCCGCCCGAGCCTGGATTACGA	TTTATTTTCAGGGCTCCATGGCGAGCGCGCGCGGCAACAAACTGCGCACCAGCCTGGAT	TTTATTTTCAGGGCTCCATGACCCGCTACAGCGTGGAACGCGCGCGCGCGAGCCTGGATCTGCCGGGCATT	TTTATTTTCAGGGCTCCATGGATGCGCCGATTGAAAAAGGCCATCGCCCGAGCCTGGATA	TTTATTTTCAGGGCTCCATGACCCGCAACACCCATGAATACGCGCGCTTCAGCCTG	TTTATTTTCAGGGCTCCATGGAACTGCTGGATTACCATTTCGAACGCAAAAGCTACGATAGCG
C	TTTATTTTCAGGGCTCCATGGGCTACACCGCGGAACAGCAGAGCCGCGCGAGCTACGATGCGCAT	TTTATTTTCAGGGCTCCATGGGCGAAAGCAGCAAAGATTTCATTCGCCTGAGCCTGGATG	TTTATTTTCAGGGCTCCATGGCGGCGCTGGGCGCGATTAAAAGCCGCCATAGCCTGGATA	TTTATTTTCAGGGCTCCATGAACAGCTTCCTGACCGATAACCTGCGCAACAGCCTGGATC	TTTATTTTCAGGGCTCCATGCAGCCGGAATTCGTGACCGATCCGCGCCTGAGCCTGGATAGCT	TTTATTTTCAGGGCTCCATGCGCTGCTACGATCGCTGGATTAACCGCGAAAGCCTGGATA
D	TTTATTTTCAGGGCTCCATGATTACCCCGACCCCGGCGTTCCAGCGCCGCAGCTACGATA	TTTATTTTCAGGGCTCCATGAAAATTAGCGATAACTTCGAACGCCGCTGGAGCTACGATA	TTTATTTTCAGGGCTCCATGTGCACCTACTGCGGCGTGTTCCGCCGCCAGAGCCTGGATCGCGGCGCGGCGAAACT	TTTATTTTCAGGGCTCCATGCAGTTCCTGCCGCCGGATGATCCGCGCAGCAGCCTGGATTGGGA	TTTATTTTCAGGGCTCCATGAGCGAACTGCGCGATCAGATTGATCGCCCGAGCCTGGAT	TTTATTTTCAGGGCTCCATGGGCGTGCCGGGCAGCGAAAACAAACGCGGCAGCCTGGATA
E	TTTATTTTCAGGGCTCCATGGAACATGATCTGTACCTGCCGCAGCGCCTGAGCCTGGATGGCGCGACCGGCACCG	TTTATTTTCAGGGCTCCATGAAACATGCGAAAACCGTGTTCAACCGCATTAGCTACGATGG	TTTATTTTCAGGGCTCCATGCTGCTGCCGAGCGGCGAAAGCTTCCGCCTGAGCCTGGATA	TTTATTTTCAGGGCTCCATGAAACATGCGAAAACCGTGTTCCAGCGCGTGAGCTACGATG	TTTATTTTCAGGGCTCCATGTACGGCATGAAATGCGATTACACCCGCTACAGCCTGGATG	TTTATTTTCAGGGCTCCATGATGAAATACCATAACCTGAGCCTGCGCCATAGCTACGATC
F	TTTATTTTCAGGGCTCCATGAACGAAGAACAGGCGAGCCATCTGCGCCATAGCCTGGATA	TTTATTTTCAGGGCTCCATGAGCGAAGAACAGGAAATGGAACTGCGCCGCAGCCTGGATGATT	TTTATTTTCAGGGCTCCATGCTGAACAGCACCAGCGTGACCCCGCGCGCGAGCCTGGATTACGA	TTTATTTTCAGGGCTCCATGCATACCCTGATGAACGTGCTGAGCCGCAACAGCCTGGATG	TTTATTTTCAGGGCTCCATGAACACCTACCCGAAACTGCTGTACCGCAACAGCCTGGATG	TTTATTTTCAGGGCTCCATGGGCCGCATTATTAGCACCATGAGCCGCCGCAGCCTGGATGCGGGCCTGAACCG
G	TTTATTTTCAGGGCTCCATGCCGGAACATATTGATGAAATTTTCCGCATGAGCTACGATTTC	TTTATTTTCAGGGCTCCATGGGCGAACATGATGCGAGCATTACCCGCAGCAGCCTGGAT	TTTATTTTCAGGGCTCCATGAGCAACAGCAGCGTGGTGCCGGTGCGCGTGAGCTACGATG	TTTATTTTCAGGGCTCCATGTTCAGCCTGGATGAAGAACTGCATCGCGTGAGCCTGGATG	TTTATTTTCAGGGCTCCATGTTCAACCAGAAAAAAAGCCTGAAACGCAACAGCTACGATGG	TTTATTTTCAGGGCTCCATGGATGATTACATGATGAACATGAAACGCAACAGCCTGGATG
H						

b

Reverse primers

	1	2	3	4	5	6
A	AATTCGGATCCCCGATATCCTTAGAAGTAGCGCGCGCTTTCGTTATCATCCAGGCTCGGGCGTTT	AATTCGGATCCCCGATATCCTTACAGGTTCACTTCCTGGAAGTTCAGATCCAGGCTCTGGCG	AATTCGGATCCCCGATATCCTTACGCTTCGCGGCGCACGCGTTCCAGATCCAGGCTCTGGC	AATTCGGATCCCCGATATCCTTACCACCACACTTTCACATGATCATGATCCAGGCTAATGCG	AATTCGGATCCCCGATATCCTTACATGCCCTGTTTGTTGCTGCTTTTATCCAGGCTGTTGCGGCG	AATTCGGATCCCCGATATCCTTAGCTCAGGAAATCGCTATCCATGTTATCCAGGCTGGTGCGGTA
B	AATTCGGATCCCCGATATCCTTAGGTCAGTTTTCTTCCCATTCCGGATCCAGGCTCCAGCGGAA	AATTCGGATCCCCGATATCCTTAGCTCACGCGGTAGCTATCAATGCTATCCAGGCTGCTGCG	AATTCGGATCCCCGATATCCTTACGCGCCCTGTTCCAGGTTATCGTAATCCAGGCTCGGGC	AATTCGGATCCCCGATATCCTTACGCAATCATCTGTTCGCGGGTGCGATCCAGGCTGCTGCGCACGTTGGTGCT	AATTCGGATCCCCGATATCCTTACGCCGCGTTGGTCACGCCCGGCAGATCCA	AATTCGGATCCCCGATATCCTTAGCTCATGCGGTAGCTGCCAATGCTATCCAGGCTGCCGC
C	AATTCGGATCCCCGATATCCTTAGTTGTTGTTGCCATGGAAGTTTTTATCCAGGCTGCTGCGAAT	AATTCGGATCCCCGATATCCTTAGTAGTTCGGCAGGAAGAACAGTTCATCCAGGCTGCTGCGCAGA	AATTCGGATCCCCGATATCCTTATTTCAGGTTGTTCAGCAGGCGCATATCCAGGCTGCTGCGATC	AATTCGGATCCCCGATATCCTTAATGATCCAGGCAATCTTTCACCGGATCCAGGCTGTTGCGGTT	AATTCGGATCCCCGATATCCTTAAATGGTGTTTTTTTTGCTCAGAATATCCAGGCTATGGCGTTTG	AATTCGGATCCCCGATATCCTTACATGTTAATCAGGTTATGATCATCATCCAGGCTCAGGCGTTT
D	AATTCGGATCCCCGATATCCTTACAGGCTTTTGCCCAGCATCTGGCCATCCAGGCTCG	AATTCGGATCCCCGATATCCTTAGCGCACGCTCGGGCTTTCGCTGGTATCCAGGCTGTAGCGGGT	AATTCGGATCCCCGATATCCTTAAATTTTGCGCGCCCAGCCGAAGTTATCCAGGCTCAGGCGGTT	AATTCGGATCCCCGATATCCTTAGGTGCAGCTTTCGCCCGCGGTGCTATCCAGGCTCAGGCGTTC	AATTCGGATCCCCGATATCCTTACAGGCGGCAGCTGTACAGGCTCACATCGTAGCTT	AATTCGGATCCCCGATATCCTTAGTAGAAGGTTTTCGGCAGCGCTTCATCCAGGCTCACGCGGGTA
E	AATTCGGATCCCCGATATCCTTAAATGTAATCGCTCGGTTTCATAATATCGTAGCTCGGGCGGGT	AATTCGGATCCCCGATATCCTTATTTGCTTTCATCGCGTTTCGGATCATCCAGGCTATGGCGATC	AATTCGGATCCCCGATATCCTTATTCCAGATCGCTGTAATCCAGAATATCCAGGCTGTAGCGATG	AATTCGGATCCCCGATATCCTTAGCCCGGCAGCAGTTTGGTGTTTTTATCCAGGCTATGGCGAATG	AATTCGGATCCCCGATATCCTTAGCCCGCCGGGTAGTTGGTTTTCGCATCCAGGCTAATGCGTT	AATTCGGATCCCCGATATCCTTAGCCGGTGCCTTTAATTTTAATGTTATCCAGGCTCAGGCGCAG
F	AATTCGGATCCCCGATATCCTTAATCTTTGAAGGTGCGCTGCGCTTCATCGTAGCTAATGCGCTG	AATTCGGATCCCCGATATCCTTACGGCGGCGGGCCCAGGCTGTAGCGATCGTAGCTAATGCGCGG	AATTCGGATCCCCGATATCCTTACAGCAGGAAGCCCTGGCTGTACAGATCCAGGCTGCAGCGCAT	AATTCGGATCCCCGATATCCTTACAGCTGCTGGTACTGGCTGCGCAGATCCAG	AATTCGGATCCCCGATATCCTTAGTTGCTCGGGAATTTCACATCGCTATCCAGGCTCAGGCGGTT	AATTCGGATCCCCGATATCCTTAATCCACCAGAATGGTTTTGCTATCATCCAGGCTGAAGCGGCT
G	AATTCGGATCCCCGATATCCTTAAATTTTGCGCACGGTCGGTTTGTAATCCAGGCTGCGGCGGAA	AATTCGGATCCCCGATATCCTTACACATCGCGCAGCTGGGTCATTTCATCCAGGCTATCGCGCAC	AATTCGGATCCCCGATATCCTTACAGGTTAATTTCGCGAATAATTTCATCGTAGCTTTCGCGTTC	AATTCGGATCCCCGATATCCTTAGAAGCTGCGGCGCGGGGTAATGCTATCCAGGCTCAGGCGGCT	AATTCGGATCCCCGATATCCTTATTCGCGCACCATGGTCAGTTCGGTATCCAGGCTAATGCGCAC	AATTCGGATCCCCGATATCCTTAGGTGGTCAGATGTTTCGCGCTAATATCCAGGCTCAGGCGCCA
H	AATTCGGATCCCCGATATCCTTAGCTGCTTTTTTCGCTGCCCAGCATATCCAGGCTGGTGCGCAC	AATTCGGATCCCCGATATCCTTACACTTTAATCACTTCCAGGAAGCTATCCAGGCTCAGGCGGCT	AATTCGGATCCCCGATATCCTTAGCTTTTGCTGCTGCTGCTGAAGTTATCCAGGCTCGCGCG	AATTCGGATCCCCGATATCCTTAGCGTTCGCCGCTGGTGCGCACCACATCCAGGCTGCGGCGTTT	AATTCGGATCCCCGATATCCTTAGCGGGTGTAGTTCAGGGTGTAATCATCGTAGCTGTTGCGGCT	AATTCGGATCCCCGATATCCTTACACGCGAATCATTTCGAAGAAGCTATCCAGGCTCAGGCG

	7	8	9	10	11	12
A	AATTCGGATCCCCGATATCCTTAGGTTTTGCTCAGGCGGCCGGTGGTATCCAGGCTATCGCGCAT	AATTCGGATCCCCGATATCCTTAATGCAGCAGGCTGCTGCTCGCCGCATCGTAGCTCGCGCGGTA	AATTCGGATCCCCGATATCCTTATTCCGGGTTCATCAGTTTCTGTTCATCCAGGCTCAGGCGTTC	AATTCGGATCCCCGATATCCTTAGCTCTGCAGAATATCCTGCAGGAAATCCAGGCTCAGGCGTTT	AATTCGGATCCCCGATATCCTTACAGGTTCAGCTGCACATCTTCGTTATCCAGGCTATCGCGCAC	AATTCGGATCCCCGATATCCTTAGCGTTTGTAGAAGCTGCTTTTCTGATCCAGGCTTTCGCGCAC
B	AATTCGGATCCCCGATATCCTTACATGGTCTGGTTCAGGTTTTCGTAATCCAGGCTCGGGC	AATTCGGATCCCCGATATCCTTACGCCAGCATTTCTTCGCGGGTGCGATCCAGGCTGGTGCGCAGT	AATTCGGATCCCCGATATCCTTATTCCGCATGGTTAATGCCCGGCAGATCCAGG	AATTCGGATCCCCGATATCCTTAGCTGTTGCGGCTTTTCGGGAAGTTATCCAGGCTCGGGCGATG	AATTCGGATCCCCGATATCCTTATTCGCTATCTTCTTCCGCCAGCGGATCCAGGCTGAAGCGCGCGTAT	AATTCGGATCCCCGATATCCTTACACGCTCAGCACGTTAATCACGCTATCGTAGCTTTTGCG
C	AATTCGGATCCCCGATATCCTTAGCCGGTATGGCCGGTTTTATGCGCATCGTAGCTCGCG	AATTCGGATCCCCGATATCCTTAGGTGTTCACGTAGGTGGTCACATCATCCAGGCTCAGGCGAAT	AATTCGGATCCCCGATATCCTTACTGCTGCTGCTGATCGCCCGGGCTATCCAGGCTATGGCGGCT	AATTCGGATCCCCGATATCCTTACAGCGGGTTATCCGCCTGGTTCAGATCCAGGCTGTTGCGCAG	AATTCGGATCCCCGATATCCTTACACGCGAATCATTTCGAAGAAGCTATCCAGGCTCAGGCG	AATTCGGATCCCCGATATCCTTAATCGTAATCGTTGCCGGTCAGAATATCCAGGCTTTCGCGGTT
D	AATTCGGATCCCCGATATCCTTATTTGAAGCTGCTGTTCATGCTAATATCGTAGCTGCGGCGCTG	AATTCGGATCCCCGATATCCTTAGTTGGTCAGTTCTTTTTTCATTTTATCGTAGCTCCAGCGGCG	AATTCGGATCCCCGATATCCTTAAATGCCCAGTTTCGCCGCGCCGCGAT	AATTCGGATCCCCGATATCCTTAGTACAGCTGGCTCTGCAGATCCCAATCCAGGCTGCTGC	AATTCGGATCCCCGATATCCTTAAATCGCCGCTTTTTCTTTCAGCACATCCAGGCTCGGGCGATCA	AATTCGGATCCCCGATATCCTTAGAAGTAATCGAACACATGTTCGCTATCCAGGCTGCCGCGTTT
E	AATTCGGATCCCCGATATCCTTAGCTTTCATCGGTGCCGGTCGCGCCATC	AATTCGGATCCCCGATATCCTTAGCATTTCACAATGCTGGTTTTGCCATCGTAGCTAATGCGGT	AATTCGGATCCCCGATATCCTTAAATAATGCCGCCTTTGCTCGGGCTATCCAGGCTCAGGCGGAA	AATTCGGATCCCCGATATCCTTAGCATTTCACAATGCTGGTCTGGCCATCGTAGCTCACGCGCTG	AATTCGGATCCCCGATATCCTTAATCGTTCCAATCCGGCAGCTGTTCATCCAGGCTGTAGCGGGT	AATTCGGATCCCCGATATCCTTATTTATCCGCGCGGCTTTTCAGCAGATCGTAGCTATGGCGCAG
F	AATTCGGATCCCCGATATCCTTAATCGGTCGCCTGCGCCACGAAGTTATCCAGGCTATGGCGCAG	AATTCGGATCCCCGATATCCTTAATCGGTCGCCTGGCTAATGTAATCATCCAGGCTGCGGCG	AATTCGGATCCCCGATATCCTTAGGTGTTCGGCACGCTCGGATCGTAATCCAGGCTCGCGC	AATTCGGATCCCCGATATCCTTAGTATTTGCTGCATTTCACGTTTTCATCCAGGCTGTTGCGGCT	AATTCGGATCCCCGATATCCTTACACGGTCAGGAACAGAATCAGATCATCCAGGCTGTTGCGGTA	AATTCGGATCCCCGATATCCTTAGTATTCCCAGCGGTTCAGGCCCGCATCCA
G	AATTCGGATCCCCGATATCCTTATTTGCTGCTGCGCTGTTCCAGGAAATCGTAGCTCATGCGG	AATTCGGATCCCCGATATCCTTAGCTCGGGTTAATGGTGCCTTTGCGATCCAGGCTGCTGCGGGTA	AATTCGGATCCCCGATATCCTTACACGGTGCTGGTGCTGCTGCTCGCATCGTAGCTCACGCGCAC	AATTCGGATCCCCGATATCCTTACACTTTAATATCGTTTTTTTTATCATCCAGGCTCACGCGATG	AATTCGGATCCCCGATATCCTTACTGTTCTTCGTTTTTTTTCAGGCCATCGTAGCTGTTGCGTT	AATTCGGATCCCCGATATCCTTATTCAATTTCTTCGGTGCCTTCCACATCCAGGCTGTTGCGTTT
H						

Supplemental table 1: **List of primers used for the Fpk1 substrate library. a**, Forward primers organized following the order found in Supp. Fig1a. The consensus motif sequence is surrounded by 5 codons unique for each peptide. **b**, Corresponding reverse primers.

Supplemental table 2

Protein	Function	Size (aa)	Motif	Putative Phospho-S Position	Detected S-Phospho
AFR1	Shmoo formation	620	qppprsskRpSLDdnesaryf	472	YES
AKL1	Endocytosis	1108	ssvstrqpRqSLDlnfqevnl	960	YES
			mrksfaraRqSLDlervrrea	1072	YES
AMS1	Mannose metabolism	1083	lpkfydkkRiSLDhdhvkvww	48	NO
ATG13	Vesicles traficking/Autophagy/TOR pathway factor	738	stsafnsrRnSLDkssnkqgm	581	YES
BAS1	Purine/histidine metabolism	811	sdtfnpeyRtSLDnmdsdfls	513	NO
BEM2	Cytokeleton organisation	2167	patpnqkmRdSLDttgrlskt	1046	YES
BPL1	Biotinylation	690	sfqfyrqyRaSYDaasssllh	327	NO
COG6	Vesicles traficking	839	kvleenkeRlSLDeqklmnpe	179	NO
COG8	Vesicles traficking	607	ddlteeqkRlSLDflqdilqs	21	NO
CTR9	Transcription	1077	aleilrkvRdSLDnedvqlnl	727	NO
CYK1	Budding	1495	afsrgalvReSLDqkssfykr	706	NO
DIT2	Sporulation	489	laemlkqfRwSLDpeweeklt	459	NO
DNF1	Lipid transport	1571	ktvgtsqpRsSLDsidsyrvs	348	NO
			yrvsadygRpSLDydnleqga	365	YES
			grgpstnvRsSLDrtreqmia	1526	YES
			nrysveraRtSLDlpgvtnaa	1552	YES
DNF2	Lipid transport	1612	rnsrsfgpRgSLDsigsyrms	386	YES
			yrmsadfgRpSLDyenlnqtm	403	YES
			asargnklRtSLDrtreemla	1566	YES
			trysveraRaSLDlpginhae	1592	YES
DNF3	Lipid transport	1656	dapiekghRpSLDnfpksrns	651	YES
ENT4	Trafficking	247	trntheyaRfSLDplaeedse	213	NO
ERG1	Ergosterol metabolism	496	clldyhfeRkSYDsvinvlsv	386	NO
ESC1	Telomeres	491	epekddiiRsSLDknfhgnnn	477	NO
GNT1	Glycans modifications	491	yldndailRsSLDelfflpny	206	NO
GPB2	PKA inhibitor	880	vhgtslmdRsSLDmrllnnlk	580	NO
GUD1	Guanine metabolism	489	ngiirfvnRnSLDpvkdcldh	56	NO
HCS1	DNA synthesis	683	arlldsnkRhSLDilskknti	298	NO
HUL5	Ubiquitin/Proteasome	910	mfialdkkRlSLDddhnlinm	532	NO
IRA2	cAMP metabolism	3079	gytaeqqsRaSYDahktghtg	547	NO
IRC3	DNA synthesis	689	gesskdfiRlSLDdvttyvnt	640	NO
JIP4	Unkown function	876	aalgaiksRhSLDspgdqqqq	752	NO
KNS1	TOR pathway factor	737	nsfltdnlRnSLDlnqadnpl	66	NO
MAL33	Maltose metabolism	468	qpefvtdpRlSLDsffemirv	267	NO
MDM30	Ubiquitin/Proteasome	598	rcydrwinReSLDiltgndyd	69	NO
MDS3	TOR pathway factor	1487	enafsatpRaSLDgqmlgksl	934	YES
MIF2	Kinetochore	549	tykrkystRySLDtsespsvr	154	YES
MKT1	Transcription regulation	830	sltsgefnRlSLDnfgwarki	719	NO
MMS22	Ubiquitin/Proteasome	1454	rsqkivteRlSLDstagesct	55	NO
MNL2	Mannose metabolism	849	tneldvtiRkSYDvslyscrl	551	NO

Protein	Function	Size (aa)	Motif	Putative Phospho-S Position	Detected S-Phospho
MTH1	Glucose sensing	433	fpnhklitRvSLDealpktfy	161	NO
MSN4	Transcription regulation	630	itptpafqRrSYDismnssfk	488	YES
MUB1	Ubiquitin/Proteasome	620	kisdnferRwSYDkmkkeltn	448	NO
NCS6	tRNA modification	359	ctycgvfrRqSLDrgaaklgi	167	NO
NTG1	DNA repair	399	qflppddpRsSLDwdlqsqly	334	NO
NUM1	Organel migration	2748	selrdqidRpSLDvlkekaai	2049	NO
NUT1	Transcription regulation	1132	gvpgsenkRgSLDsehvfdyf	958	NO
OAF3	Oleate metabolism	863	sfkinrhtRpSYDimkpsdyi	431	NO
ORT1	Arginine metabolism	292	ivkkslkdRhSLDdpkrdesk	202	NO
PIG1	Glycogen metabolism	648	pfiggedhRySLDildysdle	108	NO
PML39	RNA maturation	334	levrlksiRhSLDkntkllpg	16	NO
PRO2	Proline metabolism	456	dadlikakRiSLDaktnypag	247	NO
PRP5	RNA maturation	849	semeveelRlSLDnikikgtg	239	NO
PTR3	Amino acids sensing	678	ehdlylpqRlSLDgatgtdes	32	NO
PUS9	tRNA modification	462	khaktvfnRiSYDgktsivkc	320	NO
RAD9	DNA repair	1309	llpsgesfRlSLDspskggii	1133	NO
RIB2	tRNA modification	591	khaktvfqRvSYDgqtsivkc	293	NO
RMT2	Translation regulation	412	ygmkcdytRySLDeqlpdwnd	383	NO
SDP1	Slt2 phosphatase regulation	209	mkyhnlslRhSYDllksradk	166	NO
SEG1	Eisosome	960	rqkskthqRiSYDeaqrtfkd	145	NO
SET1	Chromatin	1080	rnslillpRiSYDryslgppp	236	NO
SKI3	RNA degradation	1432	iglvevlmRcSLDlysqgfll	818	NO
SLM2	Membrane stress	656	msyqrnsaRaSLDlrsqyqql	11	YES
SMC3	Mitosis	1230	rvtfiplnRlSLDsdvkfpsn	597	NO
SRO77	Vesicles traficking	1010	dalqlkfsRfSLDdsktilvd	566	NO
SSO1	Vesicles traficking	290	neeqashlRhSLDnfvaqatd	79	YES
SSO2	Vesicles traficking	295	seeqemelRrSLDdyisqatd	83	NO
TCB3	PI4P regulation	1545	lnstsvtpRaSLDydpsvpnt	1354	YES
TEL1	Telomeres	2787	htlmnvlsRnSLDenvkcsky	1983	NO
TEP1	PI3P Phosphatase	434	ntypkllyRnSLDdlilfltv	69	NO
TOG1	Fatty acid metabolism	794	griistmsRrSLDaglnrwey	350	NO
TRL1	tRNA modification	827	ddeietafRrSLDykptvrki	601	NO
USO1	Vesicles traficking	1790	letelknvRdSLDemtqlrdv	805	NO
VPS3	Vesicles traficking/Vacuole	1011	titvdmfeReSYDeiireinl	648	NO
VPS54	Protein recycling	889	tprarrssRlSLDsitprrsf	69	YES
VTC4	Vesicles traficking/Vacuole	721	qlpgdarvRiSLDteltmvre	375	NO
YJR098c	Unkown function	656	vqqfgydwRlSLDisakhltt	332	NO
YML35	Translation regulation	367	pehideifRmSYDfleqrssk	77	NO
YPK1	TOR pathway factor	680	gehdasitRsSLDrkgtinps	51	YES
			snssvvpvRvSYDassststv	71	YES
YPK3	TOR pathway factor	525	fsldeelhRvSLDdkkndikv	13	NO
YMR114C	Unkown function	368	fnqkkslkRnSYDglkkneeq	305	YES
YPR003c	Unkown function	754	ddymmnmkRnSLDvegteeie	572	NO
YPR078c	Unkown function	372	tllkyryvRtSLDmlgsekss	259	NO
YPR196w	Unkown function	470	qpeivtdsRlSLDsflevikv	267	NO
YTA6	Assymetric protein	754	erktnyksRaSLDnfssssks	255	YES
			kidnslikRrSLDvvrtsger	278	YES
YUR1	Protein modification	428	yfpkvklsRnSYDdytlnytr	70	NO
ZFN1	Respiratory transition	465	qpefvtdpRlSLDsffemirv	267	NO

Supplemental table 2: Table recapitulating all the peptides containing one or more RXSLD/E motif(s) in the *Saccharomyces Cerevisae* proteome. The table provides (from the left to the right) the name of the protein, its function, its length in amino acids (aa), the sequence used for cloning i.e. the consensus motif surrounded by 5 aa on both sides, the position(s) in the sequence of the serine (S) present in the consensus motif, and whether this motif was phosphorylated *in vitro*.

REFERENCE

1. Hoepfner, D., Helliwell, S. B., Sadlish, H., Schuierer, S., Filipuzzi, I., Brachat, S., Bhullar, B., Plikat, U., Abraham, Y., Altorfer, M., Aust, T., Baeriswyl, L., Cerino, R., Chang, L., Estoppey, D., Eichenberger, J., Frederiksen, M., Hartmann, N., Hohendahl, A., Knapp, B., Krastel, P., Melin, N., Nigsch, F., Oakeley, E. J., Petitjean, V., Petersen, F., Riedl, R., Schmitt, E. K., Staedtler, F., Studer, C., Tallarico, J. A., Wetzel, S., Fishman, M. C., Porter, J. A., and Movva, N. R. (2014) High-resolution chemical dissection of a model eukaryote reveals targets, pathways and gene functions. Microbiol Res 169, 107-120
